# Mediation of the total effect of cystic fibrosis‐related diabetes on mortality: A UK Cystic Fibrosis Registry cohort study

**DOI:** 10.1111/dme.14958

**Published:** 2022-09-16

**Authors:** Kamaryn T. Tanner, Rhian M. Daniel, Diana Bilton, Nicholas J. Simmonds, Linda D. Sharples, Ruth H. Keogh

**Affiliations:** ^1^ Department of Medical Statistics The London School of Hygiene and Tropical Medicine London UK; ^2^ Division of Population Medicine Cardiff University Cardiff UK; ^3^ Imperial College London, Faculty of Medicine National Heart and Lung Institute London UK; ^4^ Royal Brompton Hospital London UK

**Keywords:** cystic fibrosis, cystic fibrosis‐related diabetes, mediation analysis, pulmonary exacerbations, registries, survival analysis

## Abstract

**Aim:**

To investigate whether the effect of cystic fibrosis‐related diabetes (CFRD) on the composite outcome of mortality or transplant could act through lung function, pulmonary exacerbations and/or nutritional status.

**Methods:**

A retrospective cohort of adult cystic fibrosis (CF) patients who had not been diagnosed with CFRD were identified from the UK Cystic Fibrosis Registry (*n* = 2750). Rate of death or transplant was compared between patients who did and did not develop CFRD (with insulin use) during follow‐up using Poisson regression, separately by sex. Causal mediation methods were used to investigate whether lung function, pulmonary exacerbations and nutritional status lie on the causal pathway between insulin‐treated CFRD and mortality/transplant.

**Results:**

At all ages, the mortality/transplant rate was higher in both men and women diagnosed with CFRD. Pulmonary exacerbations were the strongest mediator of the effect of CFRD on mortality/transplant, with an estimated 15% [95% CI: 7%, 28%] of the effect at 2 years post‐CFRD diagnosis attributed to exacerbations, growing to 24% [95% CI: 9%, 46%] at 4 years post‐diagnosis. Neither lung function nor nutritional status were found to be significant mediators of this effect. Estimates were similar but with wider confidence intervals in a cohort that additionally included people with CFRD but not using insulin.

**Conclusion:**

There is evidence that pulmonary exacerbations mediate the effect of CFRD on mortality but, as they are estimated to mediate less than one‐quarter of the total effect, the mechanism through which CFRD influences survival may involve other factors.

**Novelty statement**

**What is already known?**

- Among people with cystic fibrosis, those with cystic fibrosis‐related diabetes (CFRD) experience increased mortality.

- The mechanisms underlying this effect are not well understood.

**What this study has found?**

- This study found evidence that pulmonary exacerbations mediate a portion of the effect of CFRD on mortality or transplant.
- Lung function and nutritional status were not found to be significant mediators of this effect.

**What are the implications of the study?**

- There is evidence that CFRD leads to increased pulmonary exacerbations which, in turn, adversely affect survival. This highlights the importance of reducing the risk of pulmonary exacerbations.

## 1 INTRODUCTION

Cystic fibrosis (CF) is a life‐shortening disease affecting more than 10,500 people in the UK.^1^ It is caused by mutation in the CF transmembrane conductance regulator (CFTR) gene. Of the many comorbidities people with CF are at risk of, CF‐related diabetes (CFRD) is the most common. 33.9% of people with CF aged 16 or older in the UK are being treated for CFRD and prevalence of CFRD for those over 40 is estimated to be 45%–50%.^1^, ^2^ Although people with CF over 10 years old are routinely screened for CFRD so that insulin treatment can begin promptly, those with CFRD have worse outcomes.^3^, ^4^ Unlike other forms of diabetes, the primary cause of death for people with CFRD is pulmonary failure.^5^, ^6^

There is consistent evidence that CFRD negatively impacts survival^2^, ^4^, ^7^, ^8^ but the mechanisms underlying this effect are not well understood. One hypothesis is that higher airway glucose levels in those with CFRD provide favourable conditions for bacterial growth, leading to increased infection and pulmonary exacerbations.^9^, ^10^ Also, reduced elasticity of the lung due to hyperglycaemia results in diminished lung function.^11^ Increased understanding of the mechanisms through which CFRD affects survival could inform the diagnosis and management of CFRD. Mediation analysis facilitates the exploration of pathways or mechanisms through which an exposure affects an outcome. Here, we used mediation analysis to investigate how the total effect of CFRD on mortality can be separated into an indirect effect (the portion of the total effect that acts through the specified mediator) and a direct effect (the portion of the total effect that does not act through the mediator but may involve other pathways). Because CFRD is associated with poor lung function,^12^, ^13^, ^14^ increased pulmonary exacerbations^9^, ^15^, ^16^ and worse nutritional status,^12^, ^13^, ^15^ and these three conditions are also associated with increased mortality, we considered these as potential candidates for mediators of the effect of CFRD on survival.

In this retrospective cohort‐based exploratory analysis using UK CF Registry data, we aimed to investigate the extent to which the effect of CFRD on mortality or transplant is mediated through lung function, pulmonary exacerbations or nutritional status. This is the first application of causal mediation methods to study multiple candidate mechanisms in CFRD. We apply a recently proposed method designed for a setting with a survival outcome, repeatedly measured mediators and adjustment for confounding by other time‐updated covariates.^17^

## 2 STUDY POPULATION AND METHODS

### 2.1 Data source

Anonymised data for people with CF were extracted from annual review records in the UK Cystic Fibrosis Registry for visits between 1/1/2010 and 31/12/2020. The registry, administered by the Cystic Fibrosis Trust, is a Research Ethics Committee approved research database (REC ref:07/Q0104/2) holding systematically collected data on ≥99% of people with CF in the UK.^18^ Informed consent for data collection has been obtained from adults, and for children by their parent or guardian. We chose 2010 as the study period start because from then, ≥70% of adults were screened for CFRD at their annual review. Clinical parameters are collected at annual review at a time of clinical stability and entered on the Registry, including percent predicted forced expiratory volume in 1 s (FEV_1_%),^19^ CFRD status (diagnosis usually based on oral glucose tolerance test^20^) and pulmonary exacerbations (total days on IV antibiotic per year as a proxy). Total IV days were grouped into six categories: 0, 1–14, 15–28, 29–42, 43–56 and >56 days. Nutritional status was quantified by body mass index (BMI) (kg/m^2^). 4% of FEV_1_% and 2% of BMI measurements were missing; values were filled in using the last observation carried forward. Less than 3% of IV days data was missing; values were filled in with zero. Calendar year of the visit and sex (male/female) are also used in the analysis. A person with CFRD was considered to be insulin‐treated if chronic insulin therapy was initiated prior to the annual review following diagnosis of CFRD. The outcome was time to the composite of death or lung transplantation. We chose this outcome because lung transplantation is offered when patients have advanced lung disease and because disease trajectories are different post‐transplant.

To define the analysis set, we also used data on pancreatic insufficiency (defined by pancreatic enzyme supplementation) and CF genotype class, which was defined as high risk or low risk based on previously described genotype criteria.^21^ Although the spectrum of disease seen in CF cannot be explained by CFTR mutation type alone,^22^ high‐risk genotypes are associated with both an increased risk of developing CFRD^23^ and more severe disease.^21^ Pancreatic insufficiency, also associated with high‐risk genotypes, has been shown to be an independent risk factor for CFRD.^23^

### 2.2 Study population

**FIGURE 1:**
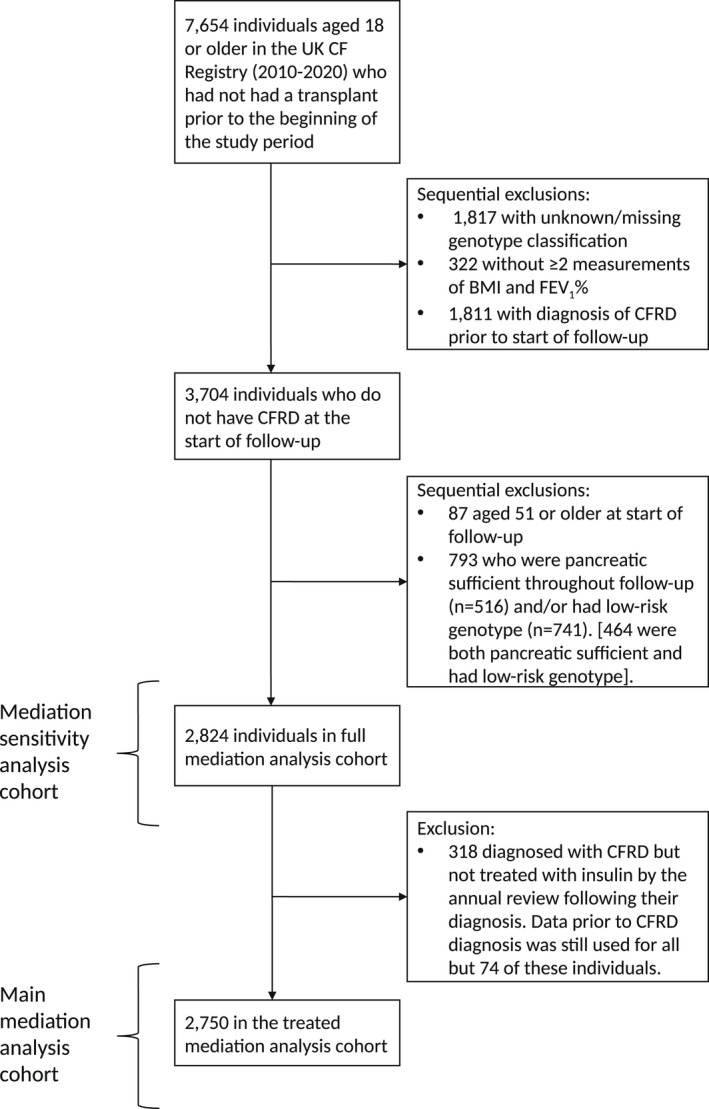
Flowchart of exclusion of individuals in the analysis populations.

The analysis cohort included individuals who, for at least some time between 1/1/2010 and 31/12/2020, were aged 18 years or more, alive and transplant‐free. We excluded those without a known genotype classification and with <2 measurements of FEV_1_% and BMI (Figure 1). Individuals who were already diagnosed with CFRD prior to their first visit during the study period were excluded to allow us to focus on incident CFRD. It is not possible to study mediators of the effect of CFRD using subgroups of patients who either all have CFRD or all do not have CFRD (called the positivity assumption).^24^ Therefore, we excluded those with low‐risk genotype and those who were pancreatic sufficient because very few had CFRD so that their effects could not be estimated in individuals with CFRD. The main mediation analysis included people who were either not diagnosed with CFRD or diagnosed with CFRD and treated with insulin. As a sensitivity analysis, the mediation analysis was repeated, this time including people diagnosed with CFRD who had not started insulin treatment.

### 2.3 Statistical analyses

#### 2.3.1 Mortality/transplant rate

The crude mortality/transplant rate was estimated as the total number of events divided by the total person‐years of follow‐up. For each person, the first visit determined the beginning of follow‐up; the earlier of date of death, date of transplant, date of last visit +2 years or 31/12/2020 defined the end of follow‐up. Poisson regression was used to estimate age‐specific mortality or transplant rates by CFRD status and by sex per 100‐person years. We performed the analysis separately by sex because life expectancy for people with CF differs by sex^1^ and some studies have reported worse outcomes for women with CFRD.^7^, ^8^

#### 2.3.2 Mediation analysis: mediators, confounders and exposure

Mediation analysis allows us to estimate the portion of the total effect of an exposure (CFRD) that acts on the outcome (death/transplant) via the mediator, which is referred to as the indirect effect. The remainder of the total exposure effect acts either directly on the outcome or via other pathways not involving the mediator (direct effect).

**FIGURE 2:**
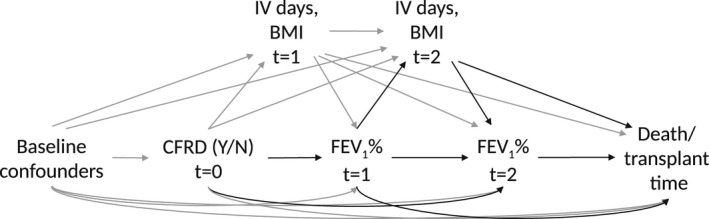
Directed acyclic graph (DAG) illustrating the temporal ordering assumed in the mediation analysis when the candidate mediator is lung function (FEV_1_%). Arrows in black indicate the combination of pathways that represent the indirect effect via the mediator. In these pathways, cystic fibrosis‐related diabetes (CFRD) first impacts the mediator. For simplicity, we only show annual review measurements taken at *t* = 1, 2 years after evaluation of CFRD but a longer follow‐up may be used.

Three potential mediators were investigated separately: lung function, pulmonary exacerbations and nutritional status. In each mediation analysis, one of these variables was selected as the candidate mediator and the other two were included as possible time‐varying mediator‐outcome confounders. We controlled for four baseline confounders of the exposure–outcome relationship: sex, calendar year (to control for differing treatments/management over the study period), FEV_1_% and BMI. Figure 2 illustrates an example of the assumed data‐generating mechanism.

For this analysis, CFRD status (Yes—exposed or No—unexposed) is noted at each annual review. At each visit, ‘baseline covariates’ refer to those measured in the previous year, that is, prior to ascertainment of CFRD status. Measures of the mediator and covariates are also available approximately annually between the recording of CFRD status and the time of the outcome or censoring. For each individual, we create an individual dataset starting at each visit (time *t* = 0), comprising CFRD status at the visit, baseline covariates measured prior to that (time *t* = −1), and mediators and outcomes measured subsequent to that (*t* = 1, 2, 3, …). CFRD status taken at t=0 is assumed fixed. This is repeated at each visit up to and including the visit at which CFRD status is ‘Yes’ or up to the last visit for people with CFRD status ‘No’, creating a sequence of datasets for each individual. Time t = 0 refers to the first visit in each sequence. For example, if visits are recorded for an individual annually at ages 20–23 and they were first diagnosed with CFRD at age 23, this individual would have four sequences used in the analysis, three starting with CFRD status ‘No’ (at t=0, age 20; t=0, age 21; and t=0, age 22) and one starting with CFRD status ‘Yes’ (at t=0, age 23). The sequences of individual datasets are combined across all individuals for the analysis.

#### 2.3.3 Mediation analysis

We implemented a mediation analysis method designed for survival outcomes that captures the indirect effect of exposure on outcome through the longitudinal mediator measurements between exposure and outcome.^17^ We estimate the total effect of CFRD on time to death or transplant, the indirect effect of CFRD that acts through the mediator and the proportion of the total effect of CFRD acting via the mediator. These effects are quantified as differences between survival probabilities calculated for three hypothetical (counterfactual) scenarios: (i) if no one had been diagnosed with CFRD, (ii) if everyone had been diagnosed with CFRD and (iii) if everyone had been diagnosed with CFRD but their mediator levels were set to levels that would have been seen if they did not have CFRD. The total effect is defined as the difference between the survival probabilities under scenarios (ii) and (i) and the indirect effect is the difference between scenarios (ii) and (iii). The percent mediated is the total effect divided by the indirect effect.

95% confidence intervals were computed at three time points using nonparametric bootstrap and the percentile method with 500 bootstrap samples. Further details of statistical methods are provided in the Supporting Information.

## 3 RESULTS

### 3.1 Study population

After excluding people with no genotype classification or <2 measurements of BMI and FEV_1_%, as well as people already diagnosed with CFRD at their first visit, 3704 individuals remained (Figure 1). Further excluding individuals who had low‐risk genotype, were pancreatic sufficient throughout the study period, were aged >51 at the start of follow‐up (because of insufficient data in older age groups) and people who were diagnosed with CFRD but not treated with insulin, there were 2750 individuals in the main mediation cohort. A sensitivity analysis cohort contained all individuals in the main mediation cohort plus 318 people diagnosed with CFRD but who had not started insulin treatment.

Characteristics of the main mediation analysis cohort at the start of follow‐up are presented in Table 1. Of the 2750 people in this cohort, 599 were diagnosed with CFRD during the study period: 101 aged 18–20 years, 316 aged 21–30, 133 aged 31–40 and 49 aged 41–50.

**TABLE 1:** Characteristics at the start of follow‐up for the main mediation analysis cohort (*n* = 2750)

Categorical analysis variables		No.	%
Sex	Female	1117	41%
	Male	1633	59%
Total IV days (prior year)	0 days	1225	45%
	1–14 days	519	19%
	15–28 days	367	13%
	29–42 days	248	9%
	43–56 days	136	5%
	>56 days	255	9%
Diagnosed with CFRD during study period		599	22%
**Continuous analysis variables**		**Median**	**IQR**
Age at start of follow‐up (years)		21.0	(18.8, 26.9)
FEV_1_%		69.9	(50.5, 84.8)
BMI (kg/m^2^)		21.5	(19.7, 23.6)
**Additional clinical information**		**No.**	**%**
F508del mutation^a^	Homozygous	1779	65%
	Heterozygous	755	27%
	Other/Unknown	216	8%
*Pseudomonas aeruginosa* ^b^	Chronic	1354	49%
	Intermittent	435	16%
	Not present	943	34%
	Unknown	18	1%

Abbreviations: BMI, body mass index; CF, cystic fibrosis; CFRD, cystic fibrosis‐related diabetes; FEV1, forced expiratory volume in 1 s.

^a^ F508del is the most common mutation amongst the UK population of people with CF.^1^

^b^ *Pseudomonas aeruginosa* is a bacterium that frequently infects the lungs of people with CF.

### 3.2 Mortality rate analysis

**FIGURE 3:**
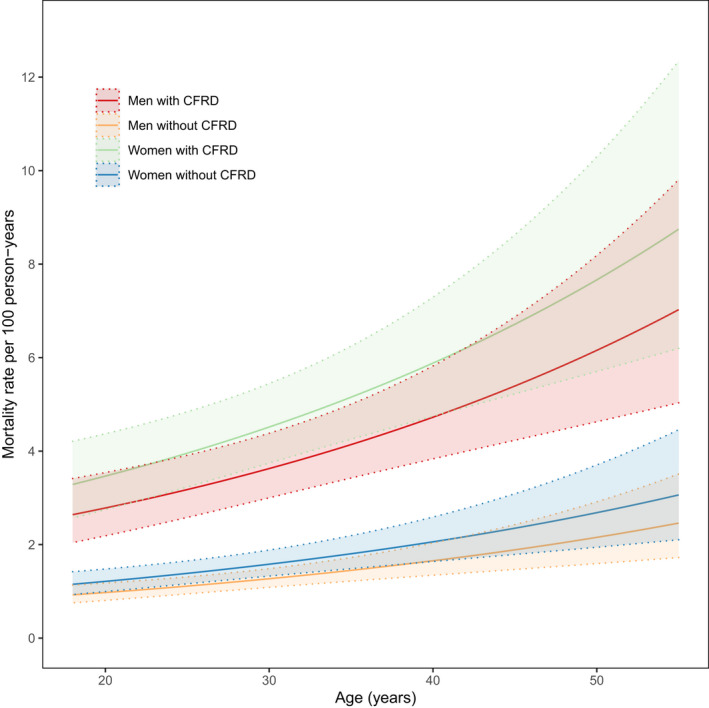
Estimated mortality/transplant rates for adults with cystic fibrosis by age, sex and cystic fibrosis‐related diabetes (CFRD) status for the mediation cohort. Shaded areas represent 95% confidence intervals.

There were 383 composite events during the study period, of which 260 (68%) were deaths and 123 (32%) were transplants. The crude mortality/transplant rate during the 21,253 person‐years of follow‐up was 1.8 per 100 person‐years. Figure 3 shows the estimated mortality/transplant rates by CFRD status, sex and age with shaded areas indicating 95% confidence intervals. For adults at all ages and for both sexes, CFRD is associated with a higher estimated mortality/transplant rate. The estimated mortality/transplant rate at age 30 for a man without CFRD was 1.3% [95% CI: 1.1%, 1.5%] compared to 3.6% [95% CI: 3.0%, 4.4%] for a man with CFRD. The estimated mortality/transplant rate for 30‐year‐old women with CFRD was greater at 4.5% [95% CI: 3.7%, 5.4%]. 30‐year‐old women without CFRD had an estimated mortality/transplant rate of 1.6% [95% CI: 1.3%, 1.9%]. Including interaction terms for CFRD with age or sex did not significantly improve model fit. No evidence of overdispersion or incorrect specification was found based on a $\chi^{2}$ test of deviance 173.7 [*p* = 0.49, *df* = 161].

### 3.3 Mediation analysis

**FIGURE 4:**
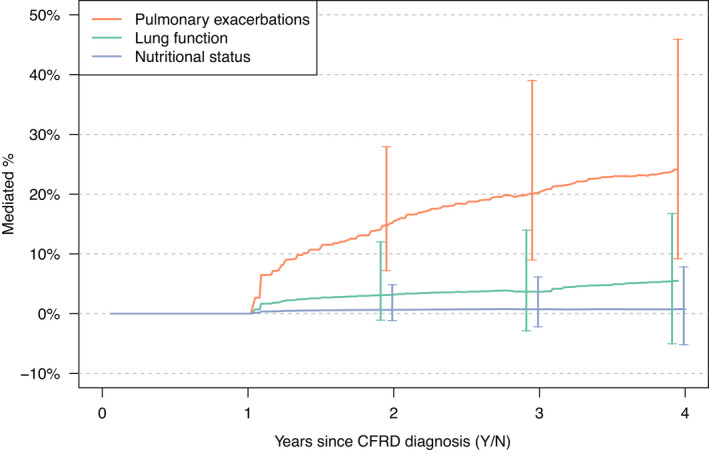
Mediation analysis results illustrating the percent of the total effect of cystic fibrosis‐related diabetes (CFRD) on mortality/transplant that is mediated by pulmonary exacerbations, lung function and nutritional status. The analysis population includes those not diagnosed with CFRD and those diagnosed with CFRD and treated with insulin (*n* = 2726). 95% bootstrap confidence intervals were calculated at three time points, time *t* = 2, 3 and 4 years post‐evaluation of CFRD and are shown as vertical bars.

Figure 4 plots the estimated percentage of the total effect of CFRD on death/transplant that is mediated through each of the three candidate mediators from 0 to 4 years after CFRD diagnosis. The strongest evidence was for pulmonary exacerbations being a mediator of the total effect of CFRD on death or transplant. Pulmonary exacerbations were estimated to mediate 15% [95% CI: 7%, 28%] of the effect at 2 years post‐CFRD evaluation, rising to 20% [95% CI: 9%, 39%] and 24% [95% CI: 9%, 46%] at 3 and 4 years, respectively. There was some suggestion that lung function may also mediate some of the total effect, but nutritional status was not found to be a significant mediator. Two years post‐evaluation of CFRD, the estimated percent mediated by lung function was 3% [95% CI: −1%, 12%] increasing to 5% [95% CI: −5%, 17%] at 4 years and the confidence intervals included 0% at all time points. Nutritional status mediated an estimated 1% [95% CI: −1%, 5%] of the effect at 2 years after CFRD evaluation.

**FIGURE 5:**
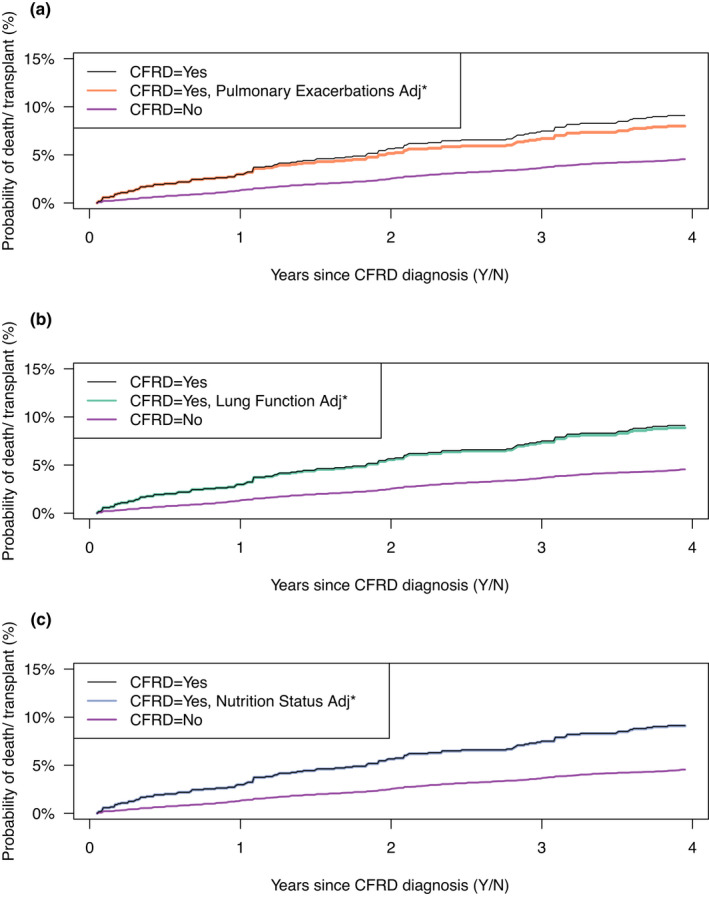
Mediation analysis results. Estimated probability of death or transplantation over time for hypothetical scenarios (i), (ii) and (iii) defined in section 2.3.3. The black line shows probabilities under scenario (i) where everyone was diagnosed with cystic fibrosis‐related diabetes (CFRD), and the purple line shows probabilities under scenario (ii) where no one was diagnosed with CFRD. The black and purple lines are the same on all three panels, and the difference between the black and purple lines represents the total effect of CFRD on mortality/transplant through all pathways. *The ‘adjusted’ lines in each panel show the counterfactual probability of death or transplantation if all individuals were diagnosed with CFRD but their mediators were set to the level they would have been at if they had not been diagnosed with CFRD (scenario (iii)). Top panel (a): For Pulmonary exacerbations as the mediator (orange line). Middle panel (b): For forced expiratory volume in 1 second (FEV_1_%) as the mediator (green line). Bottom panel (c): For Nutritional status as the mediator (blue line). The indirect effect of CFRD on mortality/transplant via each mediator is given by the difference between the black line and the orange, green or blue line for the three mediators, respectively.

To illustrate the effect of mediation, for each of the three mediators, we plot the estimated probability of death/transplant over time in Figure 5 for three hypothetical scenarios defined above: (i) if no one in the study cohort had been diagnosed with CFRD (purple), (ii) if everyone had been diagnosed with CFRD (black), and (iii) if individuals with CFRD had their levels of pulmonary exacerbations (top), lung function (middle) or nutritional status (bottom) set to the levels they would have been if they did not have CFRD. The difference between curves (ii) and (iii) represents the indirect effect of CFRD acting through the mediator, while the difference between (i) and (iii) represents the effect of CFRD that does not act through the mediator. For example, in the top graphic, the difference between the survival probabilities of scenario (ii) (black line) and scenario (iii) (orange line) is the estimated indirect effect of CFRD on death or transplant that acts through increased pulmonary exacerbations. At 4 years post‐diagnosis, the estimated indirect effect via pulmonary exacerbations was 0.01.

#### 3.3.1 Sensitivity analysis

In the sensitivity analysis that included individuals diagnosed with CFRD regardless of their insulin treatment status, we also found the strongest evidence for mediation through pulmonary exacerbations. Estimated percent mediated through pulmonary exacerbations was 20% [95% CI: 9%, 35%], 28% [95% CI: 13%, 45%] and 32% [95% CI: 16%, 53%] at 2, 3 and 4 years after evaluation of CFRD, respectively (Figure S2).

## 4 DISCUSSION

In this study, we found greater mortality/transplant rate in adults with CFRD compared to those without for both men and women. The mediation analysis suggested that pulmonary exacerbations mediate a portion of the effect of CFRD on mortality or transplant, but there was little evidence that lung function and nutritional status were significant mediators. Pulmonary exacerbations were estimated to mediate 24% of the effect at 4 years post‐CFRD diagnosis in the insulin‐treated cohort. A sensitivity mediation analysis using a cohort formed regardless of insulin treatment status found similar results.

These findings have important implications for clinical teams. Importantly, they reinforce the need to reduce the risk of pulmonary exacerbations at every opportunity, with particular focus on treatments known to be effective, including inhaled antibiotics for bacterial suppression, mucoactive therapies such as dornase alfa and more recently CFTR modulators, which have a marked effect on exacerbation risk. Although pulmonary exacerbations are well known to be associated with worse long‐term outcomes such as survival,^25^ this study adds an important extra dimension to discussions with adults with CFRD about optimal management and adherence.

This study is the first to quantitatively investigate pulmonary exacerbations, lung function and nutritional status as potential mediators of the effect of CFRD on mortality or transplant. The mediation method used^17^ allowed us to model mediators and time‐varying confounders over time, overcoming limitations of earlier methods that summarised longitudinal mediators at a single timepoint.

Using the UK CF Registry dataset, our study population was relatively large and, as it is a national, unselected dataset, it is representative of people with CF throughout the UK. Because the frequency of measurements is pre‐defined via the annual review process, there was no selection bias due to timing of the clinical visits. However, mediator measurements for lung function and nutritional status were only captured approximately annually. If physiological changes in the mediators caused by CFRD, which, in turn, affect survival, are evolving more quickly or if important variability is missed in annual measurements, this could attenuate the estimated mediation effect.^26^ Also, we used IV antibiotic days as a proxy for pulmonary exacerbations, but we acknowledge that some exacerbations may be treated with oral antibiotics and, therefore, not be captured in this analysis. Our use of the composite outcome of death or transplant is also a potential limitation because the waiting time between referral and transplant is variable and may depend on factors other than disease severity such as body size or blood group.^27^ Future research treating death and transplant as competing events could alleviate potential bias in the time to event due to contraindications for transplant. Another challenge with observational datasets is appropriate control for confounding and although we believe we have controlled for all important baseline and mediator–outcome confounders, this is a strong assumption.

CFRD can be difficult to diagnose both because temporary hyperglycaemia is common during acute illness and because many people with CF have fluctuating glucose levels throughout the day.^5^, ^28^, ^29^ This may result in undiagnosed or misdiagnosed people in our dataset, which could result in biased estimates. Additionally, the exact date of diagnosis was not available, and it is recognised that there is some treatment variation between centres with some introducing insulin earlier than others, particularly if clinical instability is present. A separate but possibly related issue is that both lung function and nutritional status have been found to decline up to 4 years prior to diagnosis with CFRD.^12^ If these declines are caused by pre‐diagnosis CFRD‐related processes, then an analysis considering diagnosis of CFRD as the starting point of physiological changes will underestimate the mediation or possibly fail to capture it.

The physiological pathways through which CFRD affects mortality may take many years to be realised but our analysis was limited to 4 years post‐diagnosis of CFRD and so did not capture longer‐term mediation. Due to limited data in later years, the mediation estimates have wide confidence intervals. Also, we treated diagnosis of CFRD as a fixed exposure for each person at each age but many people who did not have CFRD initially went on to develop CFRD later. The ability to account for a time‐varying exposure would be a helpful methodological advance.

In conclusion, this mediation analysis found evidence that pulmonary exacerbations mediate a portion of the effect of CFRD on death or transplant. As less than one‐quarter of the total effect was mediated through exacerbations, this leaves an open question about what other mechanisms exist to explain the effect of CFRD on mortality. A related question is whether the search for these mechanisms should begin at some time prior to diagnosis of CFRD when physiological changes may have already begun.

## CONFLICT OF INTEREST

NJS reports receiving honoraria for speaking engagements from Vertex, Gilead, Chiesi and Teva. He also reports honoraria for advisory boards from Vertex, Chiesi, Gilead and Menarini.

## Supporting information


Data S1
