# Supplementary material for: Mediation of the total effect of cystic fibrosis‐related diabetes on mortality: A UK Cystic Fibrosis Registry cohort study
Source: Diabet Med. 2022 Sep 16;39(11):e14958. doi: 10.1111/dme.14958 (PMC9826418; doi:10.1111/dme.14958)
Supplement: Supplementary file 1 — Data S1 [file DME-39-0-s001.docx]

Supplementary Information

Mediation of the total effect of cystic fibrosis-related diabetes on mortality: A UK Cystic Fibrosis Registry cohort study

Kamaryn T Tanner, Rhian M Daniel, Diana Bilton, Nicholas J Simmonds, Linda D Sharples*, Ruth H Keogh*

*Joint senior author

# Mortality rate analysis model

Age-specific mortality rates were estimated by sex and CFRD status per 100 person-years. A person-years table was constructed from the 22,618 annual review records in the incident cohort. Each individual contributes to the person-years table at each year of age. For example, a man first observed at age 33.6 who was diagnosed at age 35.1 and died at age 36.9 would contribute without CFRD to the person-years table at ages 33, 34 and 35 and with CFRD to the table at ages 35 and 36. An event would be recorded at age 36.

To model mortality rate, we use a Poisson regression with the addition of an offset term equal to log-person years. We write:

$$\log\left( u_{i} \right)=\log\left( t_{i} \right)+\beta_{0}+\beta_{\text{CFRD}}*\text{CFRD}_{i}+\beta_{\text{sex}}*\text{sex}_{i}+\beta_{\text{age}}*\text{age}_{i}$$

where $i$ indexes the row of the person-years table. Age was centred at 30 years. Interaction terms between CFRD with age and CFRD with sex were not significant nor was a quadratic age term.

# Mediation analysis methods

## Estimands

We applied the mediation method described by Vansteelandt et al.^1^ for time-to event outcomes and repeatedly measured mediators and confounders. In this nested counterfactual framework, the effect of the exposure on the outcome is inferred using the combination of path-specific effects via the time-varying mediator measurements. Our estimands are:

- $S_{\text{CFRD=Y, M(CFRD=N) }}(t)$, the counterfactual probability of survival to time $t$ for an individual if they were diagnosed with CFRD (at time $t=0$) but their mediator levels were set to the levels they would have been at if the individual had not been diagnosed with CFRD
- $S_{\text{CFRD=Y, M(CFRD=Y)}}(t),$ the probability of survival to time $t$ for an individual if they were diagnosed with CFRD (at time $t=0$)and their mediator levels were left at the levels we would have seen with CFRD
- $S_{\text{CFRD=N, M(CFRD=N)}}(t),$ the probability of survival to time $t$ for an individual if they were not diagnosed with CFRD (up to time $t=0$) and mediator levels were set to the levels we would have seen without a diagnosis of CFRD

Contrasts between these three survival curves can be used to estimate the indirect and total effects at time $t$:

Indirect effect$\left( t \right)=S_{\text{CFRD=Y}\text{, M(CFRD=Y)}}\left( t \right)-S_{\text{CFRD=Y,}\text{ }\text{M}\text{(CFRD=N)}}\left( t \right)$

Total effect$\left( t \right)={S_{\text{CFRD=Y}\text{, M(CFRD=Y)}}\left( t \right)-S}_{\text{CFRD=N}\text{, M(CFRD=N)}}\left( t \right)$

The proportion of the total effect mediated is:

Proportion Mediated$\left( t \right)=\frac{\text{Indirect effect}}{\text{Total effect}}=\frac{\left[ S_{\text{CFRD=Y}\text{, M(CFRD=Y)}}\left( t \right)-S_{\text{CFRD=Y,M}\text{(CFRD=N)}}\left( t \right) \right]}{\left[ {S_{\text{CFRD=Y}\text{, M(CFRD=Y)}}\left( t \right)-S}_{\text{CFRD=N}\text{, M(CFRD=N)}}\left( t \right) \right]}$

## Estimation Procedure

Vansteelandt et al.^1^ have shown that $S_{CFRD=a, M\left( CFRD=a^{*} \right)}(t)$, where $a, a^{*}=$Y/N and $a$ may or may not be equal to $a^{*}$, can be identified by:

$$S_{CFRD=a, M\left( CFRD=a^{*} \right)}\left( t \right)=\int\Pr(T>t|T>\underline{t},\bar{M}_{\underline{t}}, \bar{L}_{\underline{t}},CFRD=a,Z_{0})$$

$$\times\prod_{s=1}^{\underline{t}} f(M_{s}|T>s, \bar{L}_{s},\bar{M}_{s-1},CFRD=a^{*},Z_{0})$$

$$\times f(L_{s}|T>s-1 , \bar{L}_{s-1},\bar{M}_{s-1},CFRD=a,Z_{0})\times f\left( Z_{0} \right)dM_{s}dL_{s}dZ_{0}$$

In the above, $T$ is the smaller of the censoring or event time, $\bar{M}_{s}, \bar{L}_{s}$ is the history of the mediator and time-varying confounders up to time $s$ and $Z_{0}$ represents the baseline confounders. The visit time at or before time $t$ is denoted by $\underline{t}$. $L$ contains both the value of the characteristic and an at-risk indicator to reflect that the individual must survive in order to provide a measurement. The above quantity is estimated using repeated regression. Models are specified for each term and fitted by working backwards from the last visit time to the first, resulting in a predicted value between 0 and 1 at each step. At the end of the procedure, the predicted survival probabilities for each individual at time $t$ are averaged to provide an estimate of $S_{CFRD=a, Med\left( CFRD=a^{*} \right)}(t)$. We compute the estimands at time intervals of 0.01 years from $t$=0.05 to $t=$4.0.

The procedure is described in pseudo code in Estimation Procedure 1 below. Additional mathematical details of the general estimation procedure can be found in the Supplementary Materials accompanying Vansteelandt et al.^1^ R code for generating simulated datasets suitable for analysis using this method is available from <https://github.com/KamTan/MediationSimulation> with details about generating and using simulated datasets given in Tanner et al.^2^

Estimation Procedure 1

Input: $dat$, the dataset containing repeatedly measured mediators $\bar{M}_{T},$ time-varying covariates $\bar{L}_{T}$, baseline covariates $Z_{0}$, and observed survival/censoring times $T$ for each individual in the cohort

**for** $\left( a, a^{*} \right)\in\{\left( Y,N \right),\left( Y,Y \right), \left( N,N \right)\}$ **do ##** $\left( a, a^{*} \right)=\left( Y,N \right)$*estimates* $S_{\text{CFRD=Y, M(CFRD=N) }}(t)$

**for** $t\in\left\{ sequence from 0.05 to 4.0 by 0.01 \right\} \mathbf{do}$

## *Step 1: survival model*

$\mathrm{dat}_{a}$ = subset($dat$ where CFRD=$a$ and survived past visit $\underline{t})$

$f_{1}$= fit from Cox regression for survival $>t$ with data=$\mathrm{dat}_{a}$ and covariates $\bar{M}_{\underline{t}}, \bar{L}_{\underline{t}},Z_{0}$

$Q^{\underline{t}}(t)$ = predicted survival probability at time $t$ from $f_{1}$ for all individuals in $dat$ who survived

past visit $\underline{t}$

## *begin repeated regressions over previous visits*

$v_{previous}$ = most recent visit number at or prior to time $t$

**for** $v\in\{sequence from v_{previous} \mathrm{to} 1, \mathrm{by} 1$} **do**

## *Step 2a: integrate over distribution of the mediator*

$\mathrm{dat}_{a^{*}}$ = subset($dat$ where CFRD=$a^{*}$ and survived past visit $v)$

$f_{2a}$ = fit from quasi-binomial regression of $Q^{v}(t)$ on $\bar{M}_{v-1}, \bar{L}_{v},Z_{0}$ with data=$\mathrm{dat}_{a^{*}}$

$Q_{m}^{v}(t)$ = predicted value at time $t$ from $f_{2a}$ for all individuals in $dat$ who survived past visit $v$

*##Step 2b: integrate over distribution of time-varying confounders*

$\mathrm{dat}_{a}$ = subset($dat$ where CFRD=$a$ and survived past visit $v-1)$

$f_{2b}$ = fit from quasi-binomial regression of $Q_{m}^{v}(t)$ on $\bar{M}_{v-1}, \bar{L}_{v-1},Z_{0}$ with data=$\mathrm{dat}_{a}$

$Q_{l}^{v-1}(t)$ = predicted value at time $t$ from $f_{2b}$ for all individuals in $dat$ who survived past

visit $v-1$

*##Step 2c: incorporate at-risk indicator inherent in* $L$

$\mathrm{dat}_{a}$ = subset($dat$ where CFRD=$a$ and survived past visit $v-1)$

$f_{2c}$ = fit from Cox regression with data = $\mathrm{dat}_{a}$ and covariates $\bar{M}_{v-1}, \bar{L}_{v-1},Z_{0}$

$Q^{v-1}(t)$ = predicted survival probability at time $t$ from $f_{2c}$ for all individuals in $dat$ who

survived past visit $v-1$multiplied by $Q_{l}^{v-1}(t)$

## End of loop over $v$

## *Step 3: take the average*

$S_{CFRD=a, M\left( CFRD=a^{*} \right)}\left( t \right)=$ average$(Q^{0}(t))$

In Estimation Procedure 1, we use Cox regression and quasi-binomial regression with a logit link as recommended by Vansteelandt et al.^1^ but any appropriate model may be used at each step. Our analysis was run in R v4.0.2^3^. For SAS users, code for this method is available in the online supplementary materials of Vansteelandt et al.^1^ Also note that depending on the step, models are fitted on those diagnosed with CFRD or without CFRD. This is consistent with estimation in a nested counterfactual framework.

## Data Preparation

The mediation analysis dataset was constructed from the longitudinal dataset (UK Cystic Fibrosis Registry data provided on 20 July 2022) in the following way:

1. We assume that all data measurements were taken at integer-valued ages and use data from the most recent annual review prior to each individual’s birthday.
2. We construct one dataset per integer age from 18 to 50 years. For example, the age 20 dataset would contain everyone who is event-free at age 20, has data available at age 19, and either has not been diagnosed with CFRD or was diagnosed with CFRD in the past year.
3. Continuing with this example, data for the baseline covariates (sex, calendar year, FEV_1_% and BMI) for the age 20 dataset is taken from each individual’s age 19 data.
4. Data start and stop times are added taking the age for the dataset to be time zero. For example, in Figure S1 below, the first three rows on the right correspond to the age 20 dataset; the start time of 0 corresponds to age 20 and the stop time of 1 to age 21. In the next two rows, the start time of 0 corresponds to age 21 and in the final row, the start time of 0 corresponds to age 22.
5. If the individual died or had a transplant during the study period, an event indicator is set to 1 in the row in which the event time is between the start and stop times.
6. Because the exposure, CFRD, is assumed to be constant over time in the analysis, the CFRD indicator is only set to 1 (Yes) for the age-specific dataset corresponding to the age at which they were diagnosed. CFRD status is set to 0 (No) in data sets in which the individual has not been diagnosed with CFRD up to or including the starting age for the age-specific dataset, even if the person is diagnosed with CFRD at a later age. As our focus is on incident CFRD, each individual has a maximum of one dataset at which their CFRD status is 1.
7. Time-varying measures of the mediator and time-varying confounders are added to the age-specific dataset using the start and stop times to indicate the time interval over which each measurement is applicable.
8. Finally, the age-specific datasets for each integer age are vertically stacked to form the final analysis dataset.

Figure S1 illustrates this procedure for a hypothetical individual with data from age 19 to 22 years. In this example, the earliest data is available at age 19 so we use this data for baseline measurements in the age 20 dataset. Note that individuals contribute data when unexposed to multiple age-specific datasets but will only contribute once as an exposed person.


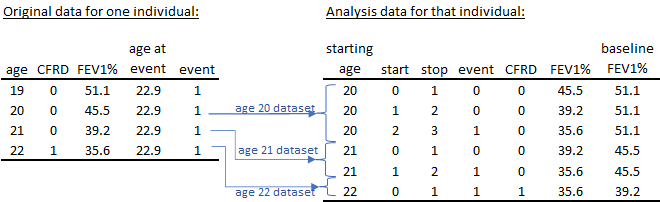


Figure S1. Construction of the analysis dataset. On the left is a subset of fictitious data for one individual after being formatted so that each record corresponds to an integer age. On the right is a table showing the three age-specific datasets (age 20, 21 and 22) created for this individual from the raw data. The CFRD indicator is a 1 (Yes) only in the age 22 dataset because that is the year in which the individual was diagnosed. In the age 20 and 21 datasets, CFRD is always 0 (No) because our analysis does not accommodate time-varying exposures.

# Mediation sensitivity analysis

Results of the mediation sensitivity analysis in the population that includes those not diagnosed with CFRD and those diagnosed with CFRD, both insulin-treated and untreated, are similar to the main analysis. Pulmonary exacerbations was estimated to mediate up to 32% [95% CI: 16%, 53%] of the total effect of CFRD on mortality at 4 years post-diagnosis. Neither lung function nor nutritional status was estimated to mediate more than 3% of the effect and 95% confidence intervals included 0% at all times.


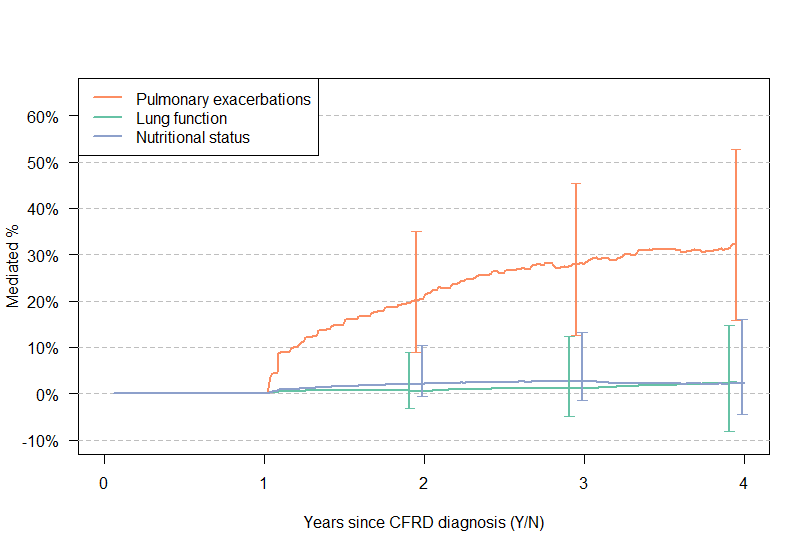


Figure S2. Mediation analysis results illustrating the percent of the total effect of CFRD on mortality/transplant that is mediated by each of the three candidate mediators: pulmonary exacerbations, lung function and nutritional status. The analysis population includes those not diagnosed with CFRD and those diagnosed with CFRD regardless of their insulin treatment status (n=2,798). 95% bootstrap confidence intervals were calculated at three time points, time $t$= 2, 3 and 4 years post evaluation of CFRD and are shown as vertical bars.

# Comparison of baseline characteristics pre-diagnosis of CFRD

Previous research investigating the pre-diabetic clinical status of people with CF found that BMI and FEV_1_% may begin to decline years prior to the diagnosis of CFRD^4^. If these declines are caused by CFRD-related mechanisms, this could lead to the mediation analysis results being understated because diagnosis of CFRD was considered the starting time. Table S1 presents a comparison of the characteristics at the start of follow-up for the 2,151 people who were not diagnosed with CFRD during the study period with the characteristics of the 599 people who were diagnosed with CFRD at some time during the study period. At the start of follow-up, none of the 2,750 had been diagnosed with CFRD. The pre-diabetic cohort was, on average, older than the controls, had a lower FEV1%, had more total IV days and was more likely to have chronic *Pseudomonas aeruginosa*. Table S1 is a crude summary of the data as we have not controlled for differing lengths of time between the start of follow-up and diagnosis of CFRD nor have we matched the characteristics such as age, sex, genotype or microbiology between the control and pre-diabetic cohort.

|  |  | Not diagnosed with CFRD | | | Diagnosed with CFRD | | |
| --- | --- | --- | --- | --- | --- | --- | --- |
| *Categorical Analysis Variables* |  | *No.* | *%* |  | *No.* | *%* |  |
| Sex | Female | 853 | 40% |  | 264 | 44% |  |
|  | Male | 1,298 | 60% |  | 335 | 56% |  |
| Total IV days (prior year) | 0 days | 1,037 | 48% |  | 188 | 31% |  |
|  | 1-14 days | 399 | 19% |  | 120 | 20% |  |
|  | 15-28 days | 278 | 13% |  | 89 | 15% |  |
|  | 29-42 days | 166 | 8% |  | 82 | 14% |  |
|  | 43-56 days | 95 | 4% |  | 41 | 7% |  |
|  | >56 days | 176 | 8% |  | 79 | 13% |  |
| Diagnosed with CFRD during study period |  | - | 0% |  | 599 | 100% |  |
| *Continuous Analysis Variables* |  | *Median* | *IQR* | | *Median* | *IQR* | |
| Age at start of follow-up (years) |  | 20.3 | (18.8, 26.2) | | 22.9 | (19.0, 28.2) | |
| FEV_1_% |  | 71.6 | (53.5, 86.4) | | 61.1 | (42.9, 77.6) | |
| BMI (kg/m^2^) |  | 21.6 | (19.8, 23.8) | | 21.2 | (19.3, 23.2) | |
| *Additional Clinical Information* |  | *No.* | *%* |  | *No.* | *%* |  |
| F508del mutation* | Homozygous | 1,368 | 64% |  | 411 | 69% |  |
|  | Heterozygous | 599 | 28% |  | 156 | 26% |  |
|  | Other / Unknown | 184 | 9% |  | 32 | 5% |  |
| *Pseudomonas aeruginosa*** | Chronic | 1,004 | 47% |  | 350 | 58% |  |
|  | Intermittent | 347 | 16% |  | 88 | 15% |  |
|  | Not present | 785 | 36% |  | 158 | 26% |  |
|  | Unknown | 15 | 1% |  | 3 | 1% |  |

# References

1. Vansteelandt S, Linder M, Vandenberghe S, Steen J, Madsen J. Mediation analysis of time-to-event endpoints accounting for repeatedly measured mediators subject to time-varying confounding. *Statistics in Medicine*. 2019;38(24):4828-4840. doi:10.1002/sim.8336

2. Tanner KT, Sharples LD, Daniel RM, Keogh RH. Methods of analysis for survival outcomes with time-updated mediators, with application to longitudinal disease registry data. *Statistical Methods in Medical Research*. Published online June 16, 2022:096228022211071. doi:10.1177/09622802221107104

3. R Core Team. *R: A Language and Environment for Statistical Computing.* R Foundation for Statistical Computing; 2020. https://www.r-project.org/

4. Lanng S, Thorsteinsson B, Nerup J, Koch C. Influence of the development of diabetes mellitus on clinical status in patients with cystic fibrosis. *European Journal of Pediatrics*. 1992;151(9):684-687. doi:10.1007/BF01957574
